# Tear Clearance Rate Assessment with a Modified Slit Lamp Biomicroscope

**DOI:** 10.1007/s44402-026-00048-w

**Published:** 2026-03-09

**Authors:** Izabela K. Garaszczuk

**Affiliations:** Visual Optics Group, Cathedral of Optics and Photonics, Department of Fundamental Problems of Technology, Wroclaw University of Science and Technology, Wrocław, Poland

**Keywords:** Dry eye disease, Fluorescein imaging, Ocular surface, Slit lamp biomicroscope, Tear clearance rate, Tear film dynamics

## Abstract

**Purpose:**

Tear clearance rate (TCR) was assessed historically with fluorophotometry and used as a marker of dry eye disease (DED). Since it requires advanced tools and specialised expertise, this method has largely been confined to research settings. This study proposed a clinically accessible method for assessing TCR using a modified slit lamp biomicroscope.

**Methods:**

This cross-sectional study included 95 subjects divided into dry eye (*n* = 65) and age-matched control (*n* = 30) groups. Five microlitres of 0.5% fluorescein solution was instilled, and tear fluorescence intensity decay was recorded over 5 min post instillation using a smartphone attached to a slit lamp biomicroscope, equipped with narrow-band filters designed to observe fluorescence. Custom-written software was used to quantify fluorescence intensity over time from the entire exposed ocular surface and calculate blinking frequency. TCR was calculated as the percentage drop in image intensity at selected intervals (0, 0.5, 1, 2, 3, 4 and 5 min) post instillation. Additionally, subjects underwent symptom assessment (ocular surface disease index (OSDI) and 5-item dry eye questionnaire (DEQ-5)), tear osmolarity, ocular surface staining, tear meniscus height, bulbar and limbal redness and noninvasive tear film break-up time (NIKBUT) assessment with the Oculus Keratograph 5M.

**Results:**

Biomicroscope-based TCRs were lower in the DED group than controls, with TCR₀.₅₋₂ (percentage decrease in fluorescence intensity between 30 s and 2 min after instillation) showing the strongest discriminatory capacity between these two groups (TCR₀.₅₋₂: 6.9% vs. 13.8%, *p* < 0.01). Early-phase TCRs correlated linearly with DED severity, OSDI, DEQ-5 and ocular surface staining (*r* from −0.21 to −0.43, all *p* < 0.05) and positively with measures of NIKBUT (*r* from 0.27 to 0.30, all *p* < 0.01).

**Conclusions:**

This study demonstrated the clinical feasibility of assessing TCR using a modified slit lamp biomicroscope. The method discriminates effectively between non-DED and DED subjects, with strong correlations with established diagnostic parameters.

Key Points

- This study introduces a method to measure tear clearance rate using a modified slit lamp biomicroscope, making an important physiological marker of dry eye disease accessible in routine clinical practice.
- Measuring tear clearance over a short 2-min period provides meaningful information about tear film health, offering a faster and more practical alternative to complex laboratory-based techniques.
- Reduced tear clearance reflects poorer ocular surface health and aligns with dry eye signs and symptoms, supporting its potential role as an objective indicator to aid the diagnosis of dry eye disease.

## Introduction

Dry eye disease (DED) is a multifactorial condition of the ocular surface caused by disrupted tear film homeostasis [1]. It significantly affects the quality of life [2] and imposes a significant economic burden globally [3, 4]. The multifactorial nature and complex aetiology of DED complicate its diagnosis, often requiring multiple tests for confirmation [5]. This leads to a delay in diagnosis and impacts patient outcomes. In the Dry Eye Workshop III by the Tear Film and Ocular Surface Society (TFOS DEWS III), experts underscored the need for a straightforward, accessible and objective method to assess the tear film for the diagnosis of DED [6].

Tear clearance rate (TCR) refers to the rate at which tears are removed from the ocular surface [7]. It depends on the level of tear secretion, fluid transudation through the conjunctiva, evaporation, drainage through the nasolacrimal system and corneal and conjunctival permeability to tears, blink quality and frequency [7–9]. In a healthy eye, these hydrodynamic phenomena are in equilibrium (homeostasis) and are regulated by the lacrimal functional unit [10]. Disruption of any of these hydrodynamic processes (e.g., increased evaporation or decreased tear secretion) can initiate the vicious circle of DED [11, 12]. TCR has been shown to serve as an indirect measure of ocular surface irritation, severity of ocular surface disease [8], Meibomian gland dysfunction [13, 14] and decreased sensitivity of the ocular surface [7, 13, 15]. TCR is reduced in subjects with symptoms of DED [16–20] and in contact lens-associated papillary conjunctivitis [21].

TCR has been assessed by tracking the washout rate of a dye from the ocular surface over time [8]. Traditionally, TCR or tear turnover rate (TTR) has been measured with a fluorophotometer [9, 22–26], requiring costly laboratory equipment and specialised training [8]. As a result, despite its high potential value in diagnosing DED and ocular surface disease [16, 26], this method has been confined to research settings [8]. Multiple studies proposed new methods to evaluate tear film dynamics and TCR [6, 27, 28], aiming to simplify the procedure using colour scales or Schirmer tests [15, 29, 30]. However, these tests are subjective and have low repeatability [8, 30]. New technologies have emerged that can be used for TCR analysis, including optical coherence tomography (OCT) [31, 32] or fluorescein profilometry [27]. Results of previous studies have shown that TCR can be evaluated qualitatively and quantitatively with more accessible clinical devices [27, 28]. A major limitation of previous methods is their lack of automation, subjective diagnosis and reliance on equipment not commonly available in clinical settings. Therefore, the objective of this study was to propose a new, clinically accessible method to assess TCR with a modified slit lamp biomicroscope, the gold standard in eye care. The TCRs were measured using custom-written software and compared between an age-matched DED group and a control group.

## Methods

The study protocol was approved by the Research Ethics Committee of the Wroclaw University of Science and Technology (Approval Number: O-22-51). The study adhered to the principles of the Declaration of Helsinki. This cross-sectional study enrolled 95 participants recruited from the general public, as well as through advertisements posted on social media platforms. Based on a clinical evaluation by an optometrist, participants were divided into two groups: individuals with signs and symptoms of DED (DED group) and non-DED individuals (control group), who presented with no signs or symptoms of DED [5]. Informed consent was obtained from all participants after explaining the nature of the study. Exclusion criteria included subjects recovering after surgery and those who were unable to refrain from using ophthalmic solutions or medications for at least 1 day prior to participating in the study. Subjects who regularly wore contact lenses were required to remove them at least 3 days before starting the study [33]. Subjects arriving directly from the outdoors were given at least 10 min to adapt to the laboratory conditions.

### Materials

**Fig. 1:**
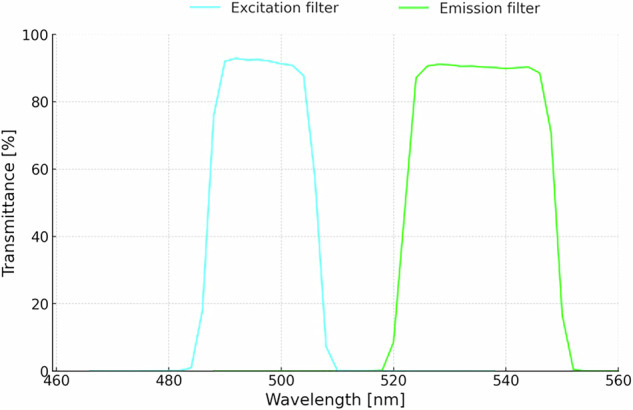
Spectral transmittance curves of the filters used in the investigation were measured prior to the study with a UV–VIS spectrophotometer.

As shown by Peterson et al., in most slit lamp biomicroscopes, the blue and yellow barrier filters are not optimal for viewing and capturing fluorescein [34]. Therefore, in this study, two edge filters, optimised for viewing yellow fluorescent protein, were used, specifically the MF497-16 (Thorlabs, thorlabs.com) excitation band filter and the MF535-22 (Thorlabs, thorlabs.com) emission band filter (with maximum transmittance of 467 ± 8 and 535 ± 11 nm, respectively). Figure 1 shows the spectral transmission characteristics of these filters assessed with the UV–VIS spectrophotometer (UV-5100, Metash Instruments, metashcorp.com). Compared to the filters provided with the biomicroscope, these filters offer higher transmission at the desired excitation wavelength (>90%), with a sharp spectral cut-off and much lower transmission at other wavelengths (<0.001%).

**Fig. 2:**
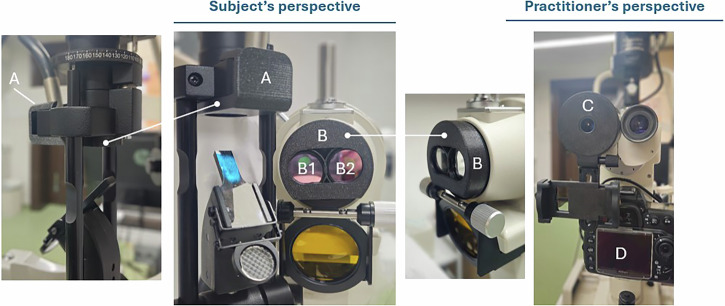
Set-up for viewing fluorescence of the tear film. The excitation band filter (**A**) was attached to the slit lamp, and the emission band filters (**B**1 and **B**2) were placed in front of the objective lenses of the microscope and secured with the cap (**B**). A smartphone attached to one of the eyepieces with the camera adaptor (**C**) or camera (**D**) mounted on the slit lamp biomicroscope could be used for image capture.

The filters were attached to the biomicroscope (Righton RS-1000, Righton, righton-oph.com) via 3D-printed covers, printed from polylactic acid (PLA), a biodegradable thermoplastic, using a PRUSA MINI 3D printer (Prusa Research, prusa3d.com). The excitation filter was attached below the light source (Fig. 2A), and two emission filters (Fig. 2B1, B2) were placed in front of the objective lens of the microscope and covered with a cap (Fig. 2B). Filters could be easily removed when not in use.

Emission filters mounted on the objective lenses of the microscope acted as mirrors; therefore, during recording, subjects could look at the reflection of their eyes in the mirror. This reduces the number of eye movements during recording and minimises the risk of reflex tearing caused by subjects’ looking directly at the light source. The light intensity of the slit lamp was set to its maximum value, since the filters used for this study blocked a large portion of the available light spectrum. Care was taken to comply with the safety limits described in the standard: *Photobiological safety of lamps and lamp systems* [35]. The magnification was set to a minimum to capture the whole exposed ocular surface. Videos were recorded with a smartphone camera (Samsung Galaxy S23, Samsung, samsung.com) attached to one of the eyepieces of the slit lamp biomicroscope with an adaptor (Fig. 2C) instead of the camera mounted on the viewing arm of the microscope (Fig. 2D), as the latter option is not always available in a clinical setting. The smartphone used in this study offered sufficient image resolution to capture the ocular surface, as well as manual camera settings. Manual Professional (PRO) mode of the smartphone camera was activated to ensure that the smartphone camera did not automatically adjust the exposure and focus. Optimal camera settings were adjusted on two subjects having very low TCR and high TCR, respectively, to ensure that the captured image was not over-saturated in both cases. The camera settings were set to ISO: 640, shutter speed: 1/30 s, focus: manual (0.8) and white balance: 4700 K. The front camera of a smartphone used in the study offered a 50 Mpix resolution with sufficient frame rate and shutter speed to record each blink.

### Fluorescein Concentration and Volume

Higher fluorescein concentrations in the tear film can cause fluorescein quenching or saturation of the device electronics [36], leading to an underestimation of TTR, as seen with 10% solutions [37]. Near-zero TTRs were observed in subjects when using higher concentrations [38]. Peterson et al. showed that quenching is observed when 1% fluorescein solution is instilled, with the tear film reaching the useful level of fluorescence around 20 s after instillation. Therefore, to optimise tear film fluorescence and fluorescein concentration, a 2% sodium fluorescein solution (Bausch and Lomb, ecp.bausch.co.uk) was diluted by adding three volumes of buffered saline to one volume of this solution in a sterile vial using a micropipette. Ultimately, approximately 5 µL of the created 0.5% fluorescein solution was instilled into the conjunctival sac with a micropipette (Pipet-Lite LTS L-10XLS+, Mettler Toledo Rainin LLC, mt.com). The conjunctival sac can hold this added volume of fluid without overflowing [39]. This concentration did not show signs of fluorescein quenching when viewed through the setup described above.

### Fluorescence Intensity Recording and TCR Calculation

**Fig. 3:**
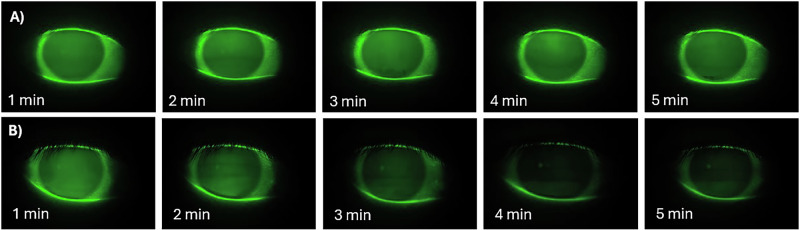
Exemplary frames sampled each minute showing the tear fluorescence intensity in subjects with: **A** low TCR and **B** high TCR. TCR tear clearance rate.

To avoid unwanted light reflections from the ocular surface, the lights in the laboratory were turned off during recording. Subjects were instructed to focus on the mirror-like surface of the emission filter. The dye was gradually replaced with new tears, and subjects were allowed to blink naturally. Fluorescence intensity was recorded for 5 min after instillation. Recording of the ocular surface allowed the observation of a decrease in the intensity of the fluorescein image (Fig. 3).

This analysis was performed under the assumption that the intensity of the recorded image is proportional to the amount of fluorescein in the subject’s tear film. The fluorescence intensity decay curve was obtained using a custom-written Python code, which calculated the mean intensity of the image within the defined area of the exposed ocular surface for every frame of the captured video. After removing blink-related artefacts, mean image intensity was measured in each subject at 0.5-min intervals from 0 to 5 min post instillation. Subsequently, TCRs were calculated as the percentage decrease in image intensity between specific time intervals, including 0, 0.5, 1, 2, 3, 4 and 5 min post instillation, which correspond to the thresholds used in previous studies of early-phase TCR [27, 28]. Statistical comparisons were made between the DED and the control group. TCRs were assessed by comparing the mean image intensity between different time points in the video recording; therefore, TCR_*X*–*Y*_ represents a percentage drop in the mean masked image intensity between the *X* and *Y*-min margin of the recording after fluorescein instillation. Negative TCRs represent a situation in which the mean masked image intensity increased over time.

### Subgroups of Subjects

Subjects were divided into DED and control subgroups based on the diagnostic methodology described by the TFOS DEWS II [5]. In summary, this consisted of a review of medical history, the ocular surface disease index (OSDI) [40] and a five-item dry eye questionnaire (DEQ-5); tear meniscus height and bulbar and limbal redness scores assessed with the Oculus Keratograph 5M (K5M; Oculus Optikgeräte GmbH, oculus.de), measurement of tear osmolarity with the TearLab Osmolarity System (Tear Lab Corp., tearlab.com) [41] and the estimation of the noninvasive keratograph tear film break-up time (NIKBUT) with the Oculus Keratograph 5M [42]. Subsequently, after a 10-min break, the fluorescein recording described above was performed using a modified slit lamp biomicroscope. After 5-min of recording, ocular surface staining was scored with the National Eye Institute (NEI) scale [43].

**Fig. 4:**
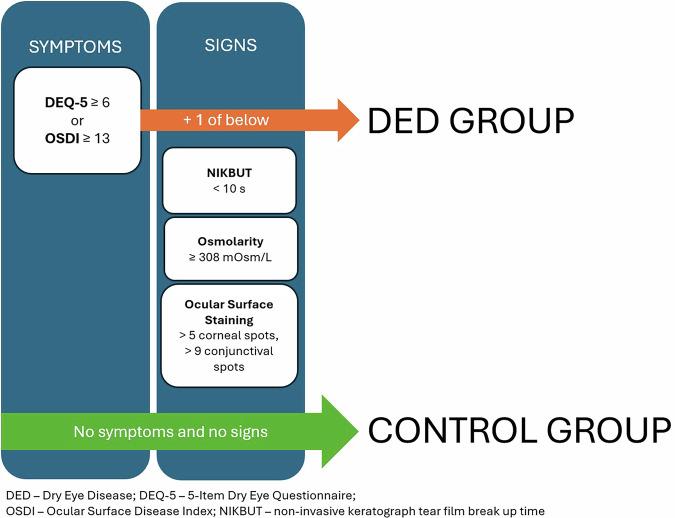
Study group subdivision protocol based on signs and symptoms of dry eye disease (DED). DEQ-5 5-item dry eye questionnaire, OSDI ocular surface disease index, NIKBUT noninvasive keratograph tear film break-up time, DED dry eye disease.

Subjects were divided into two groups based on the presence of DED markers, including symptoms (OSDI ≥ 13 and/or DEQ-5 ≥ 6) and signs (tear osmolarity ≥ 308 mOsm/L or NIKBUT < 10 s or fluorescein staining of the ocular surface staining showing >5 corneal spots or >9 conjunctival spots) [5]. The control group included subjects without visible signs or reported symptoms of DED. As summarised in Fig. 4, the DED group included subjects who had symptoms (OSDI or DEQ-5 above established thresholds) and at least one of the signs described above.

### Statistical Analysis

Statistical analyses were performed using Python (version 3.11) with custom-written scripts based on standard scientific libraries. Data normality was assessed using the Shapiro–Wilk test. Depending on data distribution, comparisons between the DED and control groups were performed using independent-samples *t* tests or Mann–Whitney *U* tests, whenever applicable.

Linear associations between TCR metrics and clinical parameters were evaluated using Pearson’s correlation coefficient (*r*). All statistical tests were two-tailed, and a *p* value < 0.05 was considered statistically significant.

### Ethical Approval

The methodology of this study was approved by the Research Ethics Committee of Wroclaw University of Science and Technology (Approval Number: O-22-51). Written informed consent was obtained from all participants.

## Results

Ninety-five subjects (72 female/23 male) aged (mean ± SD) 41 ± 14 years, range 19 to 84 years, participated in the study. They were divided into the control group (*n* = 30) and the DED group (*n* = 65). Table 1 summarises the ocular surface parameters used to allocate subjects into the subgroups. No statistically significant differences were observed for age, laboratory temperature or relative humidity (RH) in the laboratory between the DED group and control group (*p*: 0.11, 0.22 and 0.16, respectively). The laboratory temperature was (mean ± SD) 24.9 ± 1.3 °C, and the humidity was maintained at 57.2 ± 4.1% RH.

**Table 1:** Summary of ocular surface parameters used for the diagnosis of DED and *p* values for comparisons between the DED and control groups.

	Controls	DED	
	*n* = 30	*n* = 65	*p* value
	16 F/14 M	57 F/8 M	
OSDI [−]			<0.001*
Mean ± SD	3.0 ± 2.5	27.8 ± 15.9	
Range	[0.0, 9.0]	[2.1, 85.4]	
DEQ-5			<0.001*
Mean ± SD	3 ± 2	10 ± 5	
Range	[0, 5]	[0, 22]	
TMH [mm]			0.14
Mean ± SD	0.24 ± 0.07	0.23 ± 0.10	
Range	[0.12, 0.40]	[0.01, 0.64]	
Temporal bulbar redness [−]			0.27
Mean ± SD	0.8 ± 0.4	1.0 ± 0.4	
Range	[0.3, 1.6]	[0.3, 2.0]	
Nasal bulbar redness [−]			0.004*
Mean ± SD	1.1 ± 1.0	1.1 ± 0.4	
Range	[0.5, 1.7]	[0.3, 2.1]	
Temporal limbal redness [−]			0.24
Mean ± SD	0.5 ± 0.3	0.7 ± 0.8	
Range	[0.1, 1.2]	[0.1, 2.9]	
Nasal limbal redness [−]			0.06
Mean ± SD	0.4 ± 0.3	0.6 ± 0.3	
Range	[0.1, 1.1]	[0.2, 1.5]	
FNIKBUT [s]			<0.001*
Mean ± SD	15.83 ± 6.27	7.79 ± 4.74	
Range	[6.02, 24.92]	[2.29, 23.18]	
MNIKBUT [s]			<0.001*
Mean ± SD	17.16 ± 5.31	10.20 ± 4.43	
Range	[10.25, 24.92]	[3.56, 23.18]	
Osmolarity OD [mOsm/L]			0.009*
Mean ± SD	302 ± 7	307 ± 10	
Range	[290, 306]	[290, 333]	
Osmolarity OS [mOsm/L]			0.004*
Mean ± SD	300 ± 6	307 ± 10	
Range	[283, 306]	[288, 333]	
Corneal staining score (NEI)			0.002*
Mean ± SD	None	0.5 ± 1.9	
Range		[0.0, 2.0]	
Conjunctival staining score (NEI)			0.01*
Mean ± SD	0.8 ± 1.0	1.6 ± 2.4	
Range	[0.0, 3.0]	[0.0, 18.0]	

*DED* dry eye disease, *DEQ-5* 5-item dry eye questionnaire, *F* female, *FNIKBUT* first noninvasive keratograph tear film break-up time, *M* male *MNIKBUT* mean noninvasive keratograph tear film break-up time, *NEI* National Eye Institute scale, *OD* right eye, *OS* left eye, *OSDI* ocular surface disease index, *TMH* tear meniscus height.

*Denotes the statistically significant difference between subgroups.

Table 2 summarises the group means, standard deviations, medians and ranges of mean masked image intensities at different time points post instillation for DED and controls.

**Table 2:** Mean ocular surface image intensity over the recording time for the DED and control groups.

Group mean masked image intensity [a.u.]											
	Time										
[min] Group	0.0	0.5	1.0	1.5	2.0	2.5	3.0	3.5	4.0	4.5	5.0
Control group											
Mean	70.6	67.9	64.2	63.5	58.5	56.3	55.8	55.1	53.5	52.1	52.0
SD	14.8	16.6	18.3	15.9	16.3	16.7	16.5	16.8	17.1	17.5	18.5
Median	71.6	67.5	65.5	63.0	56.6	52.5	53.7	52.5	48.1	46.8	45.4
Min	46.1	40.9	35.1	36.5	31.3	29.3	31.7	33.0	27.8	25.9	25.1
Max	96.3	98.7	102.1	95.0	95.0	94.2	95.1	94.0	94.9	89.9	92.7
DED group											
Mean	73.9	73.1	72.2	69.8	69.1	67.6	66.1	63.8	63.3	62.5	61.1
SD	18.9	15.5	13.7	15.0	14.3	15.9	17.1	18.1	16.2	17.6	17.3
Median	77.7	75.3	74.5	71.2	68.6	68.4	68.6	65.0	64.9	64.4	63.6
Min	17.2	26.4	26.1	27.3	32.5	14.5	19.3	17.9	23.7	7.8	14.3
Max	100.2	97.9	95.0	97.0	95.4	97.7	97.5	93.6	92.1	92.6	92.9
*P* value	0.18	0.09	0.03*	0.09	0.004*	0.003*	0.006*	0.009*	0.01*	0.003*	0.01*

*DED* dry eye disease, *SD* standard deviation, *a.u.* arbitrary units of pixel brightness (0–255).

*Denotes statistical significance.

**Fig. 5:**
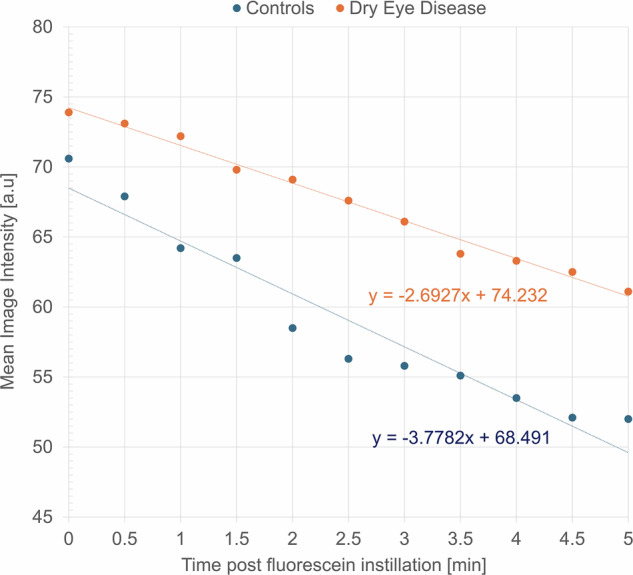
Group mean masked image intensity over time for the dry eye disease (DED) and control groups. Standard deviations (SD) were not displayed for readability. a.u. arbitrary units of pixel brightness (0–255).

In Fig. 5, the group mean image intensities over time are displayed for the DED and control groups. Statistically significant differences were evident even when the mean image intensity curves were normalised for each subject.

Table 3 summarises the TCR measured for each subgroup.

**Table 3:** TCR_*X*–*Y*_ calculated between different time margins of the recording, where *X* and *Y* represent minutes after installation.

	TCR_0–0.5_ [%]	TCR_0__–1_ [%]	TCR_0–2_ [%]	TCR_0–3_ [%]	TCR_0–4_ [%]	TCR_0–5_ [%]
Controls						
Mean ± SD	3.7 ± 14.2	9.6 ± 14.9	17.0 ± 15.9	21.3 ± 14.1	24.6 ± 15.5	26.9 ± 17.8
Median	4.4	8.9	18.5	20.7	22.4	27.9
Range	[−32.0, 31.5]	[−22.1, 36.4]	[−19.9; 49.1]	[1.3, 49.1]	[−0.1, 49.1]	[−4.4, 63.4]
DED						
Mean ± SD	4.2 ± 9.2	5.9 ± 10.7	10.1 ± 13.4	13.3 ± 18.3	17.9 ± 17.3	20.3 ± 19.6
Median	4.2	2.8	5.7	9.0	15.8	17.5
Range	[−17.7, 31.8]	[−18.0, 32.6]	[−18.2, 41.0]	[−27.4, 74.0]	[−22.7, 51.7]	[−23.9, 83.3]
*p* value	0.98	0.21	0.03*	0.01*	0.04*	0.03*

	TCR_0.5–1_ [%]	TCR_0.5–2_ [%]	TCR_0.5–3_ [%]	TCR_0.5–4_ [%]	TCR_0.5–5_ [%]	
Controls						
Mean ± SD	5.6 ± 12.7	13.8 ± 12.4	17.7 ± 12.8	21.0 ± 16.0	23.6 ± 17.1	
Median	3.9	14.9	19.7	24.6	25.6	
Range	[−34.1, 47.7]	[−19.0, 33.3]	[−15.2, 37.0]	[−21.2, 47.3]	[−8.3, 63.3]	
DED						
Mean ± SD	2.6 ± 8.6	6.9 ± 13.1	10.7 ± 16.6	14.2 ± 16.7	17.3 ± 19.4	
Median	2.2	5.0	7.7	13.9	15.1	
Range	[−28.4, 22.7]	[−26.2, 39.8]	[−25.4, 69.6]	[−32.3, 48.6]	[−26.9, 81.0]	
*p* value	0.19	0.003*	0.006*	0.06	0.08	

	TCR_1–2_ [%]	TCR_1–3_ [%]	TCR_1–4_ [%]	TCR_1–5_ [%]		
Controls						
Mean ± SD	10.0 ± 11.0	14.3 ± 10.5	18.0 ± 13.0	20.5 ± 15.3		
Median	11.9	15.6	16.2	19.5		
Range	[−36.3, 26.4]	[−12.4, 34.9]	[0.3, 45.7]	[−3.3, 62.1]		
DED						
Mean ± SD	4.8 ± 10.9	8.9 ± 14.3	13.3 ± 14.2	16.5 ± 17.3		
Median	3.3	6.0	12.0	14.9		
Range	[−26.1, 40.6]	[−20.4, 70.1]	[−24.8, 46.4]	[−25.9, 79.7]		
*p* value	<0.001*	0.004*	0.11	0.27		

	TCR_2–3_ [%]	TCR_2–4_ [%]	TCR_2–5_ [%]			
Controls						
Mean ± SD	4.3 ± 9.4	8.2 ± 14.3	11.3 ± 16.5			
Median	2.7	5.3	8.9			
Range	[−13.8, 28.9]	[−34.6, 34.1]	[−23.1, 54.0]			
DED						
Mean ± SD	4.7 ± 15.9	9.2 ± 15.6	12.6 ± 19.1			
Median	4.1	9.3	12.6			
Range	[−56.0, 69.1]	[−46.4, 38.3]	[−43.6, 76.9]			
*p* value	0.77	0.48	0.73			

	TCR_3–4_ [%]	TCR_3–5_ [%]				
Controls						
Mean ± SD	3.9 ± 9.1	7.9 ± 12.8				
Median	1.3	6.3				
Range	[−18.3, 27.5]	[−10.7, 49.4]				
DED						
Mean ± SD	5.4 ± 10.7	8.4 ± 16.3				
Median	4.9	7.9				
Range	[−25.3, 33.0]	[−61.0, 73.7]				
*p* value	0.25	0.50				

	TCR_4–5_ [%]					
Controls						
Mean ± SD	4.4 ± 9.2					
Median	3.2					
Range	[−20.4, 30.3]					
DED						
Mean ± SD	2.8 ± 15.7					
Median	2.2					
Range	[−38.7, 69.9]					
*p* value	0.40					

TCR_*x*–*y*_—tear clearance rate—percentage drop in mean image intensity between *X* and *Y* min of the recording (including 0, 0.5, 1, 2, 3, 4 and 5 min).

The *p* value corresponds to the statistical difference between the dry eye disease (DED) and the control groups.

*Denotes statistical significance.

### Linear Correlations

Linear Pearson’s correlation coefficients (*r*) were calculated for all parameters considered in Tables 1 and 3. Statistically significant linear correlations between measures of TCR and ocular surface parameters have been summarised below. Significant associations were observed between TCR metrics and multiple clinical and functional parameters. When assuming that the severity of DED is understood as a number of signs and symptoms that exceed their severe level thresholds, higher symptom severity was negatively correlated with several TCR indices, including TCR_0–2_ (*r* = −0.23, *p* = 0.03), TCR_0.5–2_ (*r* = −0.32, *p* = 0.002), TCR_1–2_ (*r* = −0.43, *p* < 0.001) and TCR_1–3_ (*r* = −0.24, *p* = 0.02), indicating reduced TCR with increasing disease severity. Patient-reported outcomes showed similar patterns, with both OSDI and DEQ scores being negatively correlated with TCR_0.5–2_, TCR_1–2_ and TCR_1–3_ (*r* ranging from −0.21 to −0.28, all *p* < 0.05). Tear film stability parameters (FNIKBUT and MNIKBUT) demonstrated positive correlations with early TCR indices (e.g., FNIKBUT vs TCR_1–2_: *r* = 0.30, *p* = 0.004; MNIKBUT vs TCR_1–2_: *r* = 0.27, *p* = 0.009), whereas ocular surface staining measures were negatively correlated with TCR_0.5–2_ and TCR_1–2_ (*r* = −0.23 to −0.26, *p* < 0.05). Additionally, specific redness parameters showed positive correlations with selected TCR ratios (e.g., bulbar nasal redness vs TCR_0–3_: *r* = 0.23, *p* = 0.03; limbal nasal redness vs TCR_2–3_: *r* = 0.26, *p* = 0.009). TCR_1–4,_ TCR_2–3_ and TCR_2–4_ were correlated with blinking frequency (*r*: 0.20, 0.29 and 0.30, respectively).

## Discussion

Despite its popularity, the fluorophotometric assessment of TTR is limited by its complexity, long duration (10–30 min) and skill requirement. The purpose of this study was to introduce a new clinically applicable technique of evaluating early-phase TCR and to test whether TCR could be used as a marker of DED. This newly proposed technique was not time-consuming (only a 2-min recording is required to estimate TCR), was easy to perform, utilised low concentration fluorescein and was performed with a commercially available clinical instrument. In addition, the large aperture of the photometric microscope reduces spatial resolution, capturing corneal tissue fluorescence along with the tear film and leading to errors in TTR estimation, especially in patients with a compromised corneal epithelium (e.g., dry eye) or treated with preserved artificial tears [9, 26, 44–47]. In addition, age-related autofluorescence must be corrected [48]. To minimise artefacts, previous studies have proposed improved protocols that require six measurements over 10 min and faster scans (~8 s) [49–51]. In contrast, long scan times (~20 s) increase blink-related errors [22], while enforced blink rates can alter blink quality and affect TTR accuracy.

The proposed slit lamp biomicroscope-based technique of TCR estimation is free from these limitations, since measurements are continuous, have higher spatial and temporal resolution, reduced reflex tearing due to intense illumination, do not require suppressed blinking conditions to perform a scan, utilise low concentrations of fluorescein so as to avoid quenching and are quick and easy to perform.

This study demonstrates that TCR can be recorded with a slit lamp biomicroscope and that TCR can discriminate between subjects with DED and a control group. As seen in Fig. 5, the mean masked image intensity curves measured in subjects with DED differed from those observed in the control group. While for the DED image intensity decay curve was linear (*y* = −2.693*x* + 74.232, *R*^2^ = 0.99), the masked mean image intensity curve in the controls had exponential or bilinear characteristics (*y* = 68.755e-0.063*x*, *R*^2^ = 0.95 vs 0.93 for linear), making the latter curve more comparable with the fluorescence intensity curves observed in non-DED subjects with fluorophotometry, OCT or fluorescein profilometry.

Furthermore, the TCRs calculated for the DED group and controls were significantly different. As can be seen in Table 3, there is a significant difference between the DED group and controls, with TCRs sampled in the 2-min margin of the recording. TCR_0.5–2_ is twice as high in the controls compared with the DED group (13.8 vs 6.9%, respectively). These findings suggest that a 2-min ocular surface recording is sufficient to diagnose DED with a slit lamp biomicroscope. Furthermore, the data summarised in Table 3 indicate that the mean image intensity curve does not need to be measured immediately after fluorescein instillation, since TCR_0.5–2_ and TCR_0.5–3_ were significantly different between the two groups. This gives the practitioner sufficient time (30 s) to instil fluorescein, adjust the slit lamp biomicroscope and start recording. The TCRs measured during the first 30 s of the recording were similar in both groups, suggesting that immediately after fluorescein instillation, tear film dynamics are largely independent of ocular surface condition. This may be explained by Krehbiel flow, i.e., the capillary-driven movement of tear fluid from the tear meniscus onto the ocular surface [52], combined with progressive redistribution of fluorescein across the ocular surface during blinking. However, it is evident that if the early-phase TCR is lower in subjects with DED, then these subjects are more likely to have negative TCRs. This may suggest that after fluorescein instillation, the tear film of DED subjects needs more time to be distributed over the ocular surface than is the case for controls. Alternatively, the concentration of fluorescein in the tear film of the DED subjects was higher. Both effects could be due to poor blink quality in DED subjects or higher concentrations of fluorescein in lesser amounts of tear film. The latter could also explain why the mean masked image intensity at the 0-min margin tended to be slightly (non-significantly) higher in the DED group. The mean intensity of the masked image remained higher in DED subjects for the whole duration of the recording, which could be explained by slower tear exchange on the ocular surface and/or higher concentrations of fluorescein in the tear meniscus. However, the tear meniscus heights of DED subjects were not significantly lower than those noted in the control group, pointing to different tear dynamics, blinking quality and fluorescein distribution as the main source of this difference, rather than differences in tear volume and fluorescein concentration. Additionally, the early measures of TCR (such as TCR_0.5–2_ and TCR_0–2_) did not exhibit a significant linear correlation with blink frequency, with TCR measures sampled at the end of the recording (including TCR_1–4,_ TCR_2–3_ and TCR_2–4_). This may suggest that early tear exchange is more dependent on blinking quality, as well as fluorescence intensity and distribution, rather than on blinking frequency.

It is important to note that in this study, measures were taken to prevent fluorescence quenching by applying fluorescein concentrations lower than typically used when dye is applied directly from a single-use vial or with a moistened paper strip. In studies where higher concentrations of fluorescein were used, an opposite trend of fluorescein image intensity could possibly be observed as a result of fluorescein quenching [36–38].

Although TFOS DEWS III was cited in the introduction to reflect the most recent consensus on DED diagnosis [6], participant classification in this study was based on the TFOS DEWS II criteria [5], as the study protocol was designed and data collection commenced prior to the publication of TFOS DEWS III. This represents a limitation of the present study. Future investigations should apply the TFOS DEWS III criteria to validate and refine the diagnostic performance of the proposed TCR assessment method further.

Another limitation of this study is that there were no standardised criteria to diagnose the severity of DED, and the diagnosis was based on the number of signs that exceeded certain thresholds from TFOS DEWS II [5]. The DED subjects were only slightly above the thresholds considered normal in most of these parameters. The majority of the DED participants had mild to moderate evaporative DED, rather than the severe, aqueous-deficient type. However, the differences between the DED and control group reported here were statistically and clinically significant, suggesting that TCR could be a marker of DED, even in mild cases. Furthermore, statistically significant linear correlations between TCR and signs and symptoms of ocular surface disease suggest that the higher the TCR, the healthier the ocular surface. TCR was significantly correlated with almost all the signs and symptoms of DED that were assessed here, suggesting its potential to become a macro-type marker of DED.

In summary, tear exchange on the ocular surface can be assessed with a modified slit lamp biomicroscope, with estimated TCRs being used to distinguish DED subjects from non-DED controls. This technique has high spatial and temporal resolution and is not limited by corneal permeability. The simplicity and size of the setting open up the possibility for miniaturisation of the device. Although early-phase TCR, particularly over the 0.5–2-min interval, showed strong discriminatory potential between DED and non-DED participants, the present study was not designed to establish diagnostic cut-off values. Validation of an optimal time-based TCR threshold will require receiver operating characteristic analysis in an independent cohort. Future studies should aim to standardise the procedure by comparing TCRs in subjects with different types and severity of DED, and by comparing biomicroscope-based values with other measures of TCR. Although differences between slit lamp biomicroscope-based TCR and TTR are apparent in terms of several characteristics, future studies should compare fluorophotometry-based TTR with biomicroscope-based TCR in an effort to standardise the newly proposed procedure.

## Data Availability

The data supporting the findings of this study are openly available in the RepOD data repository. Video recordings of the ocular surface area and custom-written Python software can be found here: Garaszczuk, Izabela, 2025, *Tear fluorescein clearance rate assessment with modified slit lamp biomicroscopy*, RepOD, V1, link: https://repod.icm.edu.pl/dataset.xhtml?persistentId=doi:10.18150/NZG1ZS. 3D templates for filter covers are openly available at: Garaszczuk, Izabela, 2025, *3D-printed covers for attaching Thorlabs optical filters to a Haag-Streit-type slit-lamp biomicroscope*, RepOD, V1, link: https://repod.icm.edu.pl/dataset.xhtml?persistentId=doi:10.18150/JKEGNE.
